# Mental time travel and insight in schizophrenia

**DOI:** 10.1017/S0033291725102572

**Published:** 2025-11-24

**Authors:** Pegah Seif

**Affiliations:** Department of Psychiatry, Beth Israel Deaconess Medical Center, Harvard Medical School, Boston, MA, USA

**Keywords:** autobiographical memory, insight, mental time travel, metacognition, schizophrenia

## Abstract

Schizophrenia features pervasive insight deficits, with many failing to recognize symptoms or the need for treatment, predictors of poorer outcomes. Rather than unitary, insight comprises clinical (awareness of illness and need for care) and cognitive (self-reflectiveness and the ability to question one’s beliefs). This review examines whether mental time travel (MTT) – vivid recollection of past events and construction of detailed future scenarios – may underlie insight deficits in schizophrenia. We synthesize evidence up to May 2025 from meta-analyses, experimental studies, and neuroimaging/neuroanatomical reports on MTT (autobiographical memory specificity, future simulation, temporal horizon) and their associations with clinical and cognitive insight. Individuals with schizophrenia show reduced autobiographical specificity, future simulation vividness, alongside a narrowed temporal horizon. These impairments are linked to diminished self-reflection, narrative coherence, and metacognitive abilities, all of which are essential for accurate illness recognition. Neuroimaging indicates that the networks supporting mental time travel, self-reflection, and insight – particularly the default-mode and ventromedial prefrontal circuits – substantially overlap and are disrupted in schizophrenia, with heterogeneity across illness stage and analytic approach. Moderators such as negative symptoms and trauma appear to intensify the MTT-insight links, while depressive mood may paradoxically enhance illness awareness. Although therapies targeting episodic specificity and metacognitive mastery show promise, longitudinal and interventional evidence remains limited. Associations between MTT impairments and insight are robust but largely correlational, so reverse or bidirectional causality cannot be excluded. We outline priorities for longitudinal, interventional, and trauma-stratified studies – attentive to illness stage and default-mode dynamics – to clarify mechanisms and guide targeted interventions.

## Introduction

Schizophrenia – a disorder affecting approximately 1% of the population – is characterized by profound disturbances in perception, thought, and self-awareness. Clinical insight deficits are prevalent, with between one-half and four-fifths of patients unable to recognize psychotic experiences as symptoms or acknowledge the need for treatment, predicting poorer medication adherence and functional outcomes (Lincoln, Lüllmann, & Rief, 2007).

Insight itself, however, is not a unitary construct (Konsztowicz, Schmitz, & Lepage, 2018). Contemporary models differentiate between clinical insight – illness acknowledgment, symptom relabeling, and recognition of treatment necessity – and cognitive insight, underpinned primarily by self-reflection, the ability to critically evaluate one’s interpretations and beliefs (David, 1990; Xu et al., 2021). Low self-reflectiveness, especially in conjunction with elevated self-certainty, consistently predicts poorer clinical insight (Cooke et al., 2010), indicating that self-reflection is necessary but insufficient alone for achieving full insight.

Critically, autobiographical memory anchors personal identity by providing vivid memories that shape the coherent sense of self (Bréchet, 2022). Extending from this foundation, mental time travel (MTT) allows individuals to vividly re-experience past events and imagine detailed future scenarios (Tulving, 1985). Meta-analytic findings highlight substantial impairments in episodic memory specificity and future simulation abilities across stages of schizophrenia (Fornara, Papagno, & Berlingeri, 2017). Such deficits undermine narrative coherence and future-directed planning, both essential components for recognizing illness and taking informed action (Mavrogiorgou et al., 2022; Stanghellini et al., 2016).

Converging neuroimaging evidence underscores a notable overlap between the neural circuits supporting MTT – particularly the default-mode and ventromedial prefrontal networks – and those engaged during self-reflection and insight processing (Lee, Parthasarathi, & Kable, 2021; Østby et al., 2012).

Because most available studies are cross-sectional, links between MTT and insight are interpreted as associations, and reverse or bidirectional explanations remain plausible.

This theory-driven narrative review synthesizes clinical, cognitive, and neurobiological evidence available up to May 2025 to elucidate the relationship between MTT and insight deficits in schizophrenia. It is proposed that impairments in MTT represent a fundamental yet understudied candidate mechanism of poor insight. By integrating existing evidence, the review highlights mechanistic pathways linking MTT, self-reflection, and insight, explores therapeutic implications, and outlines future research directions necessary to clarify this complex interplay.

## Methods – research strategy

Databases searched were PubMed, PsycINFO, Web of Science, and Scopus from inception to May 31, 2025. Search strings combined terms for mental time travel (autobiographical memory specificity, episodic future thinking, temporal horizon) AND insight (clinical, cognitive) AND schizophrenia/psychosis. Inclusion criteria were peer-reviewed human studies reporting ≥1 MTT measure and an insight outcome; exclusions were case reports, narrative reviews, and non-English articles.

## Conceptual foundations

### Clinical and cognitive insight

Early models treated insight in schizophrenia as a single construct, but extensive research indicates at least two distinct facets. *Clinical insight* involves acknowledging mental illness, identifying psychotic experiences as pathological, and recognizing the need for treatment (Lysaker et al., 2021). It is typically measured using clinician-rated tools such as the Schedule for the Assessment of Insight – Expanded (SAI-E) and Scale to Assess Unawareness of Mental Disorder (SUMD) (Amador & Kronengold, 2004; David, Buchanan, Reed, & Almeida, 1992).

*Cognitive insight* reflects a metacognitive stance toward personal beliefs. Beck and colleagues developed the Beck Cognitive Insight Scale (BCIS), which assesses *Self-Reflectiveness* (the tendency to question one’s interpretations) and *Self-Certainty* (confidence in one’s judgments) (Beck et al., 2004). Lower self-reflectiveness, especially when paired with higher self-certainty, reliably predicts poorer clinical insight, increased delusional conviction, and reduced medication adherence (Engh et al., 2007; Pedrelli et al., 2004; Riggs, Grant, Perivoliotis, & Beck, 2012). Thus, self-reflection emerges as necessary but insufficient for complete insight.

*Differential links to MTT.* In this review, clinical insight refers to clinician-rated awareness of illness/symptoms/need for treatment (e.g., SAI-E, SUMD), whereas cognitive insight refers to metacognitive self-evaluation (e.g., BCIS Self-Reflectiveness and Self-Certainty) (Beck et al., 2004). Where studies reported both, mental time travel (MTT) measures tended to show more consistent associations with clinical insight than with cognitive insight, although BCIS Self-Reflectiveness often aligns with greater episodic specificity and future-event detail (Beck et al., 2004).

*Relation to metacognition.* Cognitive insight, operationalized by the Beck Cognitive Insight Scale (BCIS), reflects a metacognitive appraisal of one’s own interpretations – indexed by Self-Reflectiveness and Self-Certainty (Beck et al., 2004). Metacognition is a broader construct that includes self-reflectivity and the ability to use that understanding to guide behavior (mastery) and to take perspectives beyond the self (decentration), as indexed by instruments such as the Metacognition Assessment Scale–Abbreviated (MAS-A) (Lysaker et al., 2005; Lysaker et al., 2021; Semerari et al., 2003). Thus, cognitive insight overlaps with the self-reflective facet of metacognition but does not encompass other metacognitive capacities (e.g., mastery), which have shown independent associations with clinical insight (Bröcker et al., 2017; Lysaker et al., 2005).

### Autobiographical memory and self-reflection

A coherent sense of self is supported by a reservoir of autobiographical memories (AM), encoding details of what happened, the context in which events occurred, and their significance for personal goals. Vivid, detailed recollections serve as reference points for evaluating current beliefs, imagining counterfactual alternatives, and updating self-knowledge (Fivush, 2011). In schizophrenia, AM is often characterized by ‘over-generalization,’ with patients recalling events only in broad, gist-like terms that lack specific temporal and spatial details – a pattern linked to impairments in executive function and affect regulation (Berna et al., 2016; Herold, Lässer, & Schröder, 2023; Mediavilla et al., 2021). Because self-reflection fundamentally relies on detailed personal evidence, a compromised AM store restricts the ability to reality-test psychotic interpretations and integrate current experiences into a coherent life narrative.

### Mental time travel (MTT)

Mental time travel (MTT) is the capacity to vividly re-experience past events and pre-experience plausible future scenarios by flexibly recombining episodic details into coherent mental scenes (Suddendorf & Corballis, 2007). Experimental paradigms reveal three consistent deficits in schizophrenia. *First*, during episodic memory tasks such as the Autobiographical Interview, patients generate significantly fewer internal details (who was present, what occurred, where, and when) compared to controls, despite similar overall verbosity (Berna, Potheegadoo, et al., 2016; Danion, Huron, Vidailhet, & Berna, 2007). *Second*, in future simulation paradigms like Scene Construction or Prospective Thinking tasks, patients typically produce vague, generic, and less vivid imagined futures, lacking concrete goals (e.g., ‘I might do something outside’) compared with detailed, goal-oriented scenarios offered by controls (e.g., ‘Next Saturday I’ll meet Tom at the café at 2 p.m. to plan our project’) (Raffard et al., 2009; Raffard et al., 2010). *Third*, temporal-horizon assessments such as the Temporal-Extension Mental Time Travel (TEMT) task indicate a marked ‘foreshortened future’ bias, with patients typically envisioning only the near-term future (days or weeks ahead), whereas controls project months or years ahead; a recent meta-analysis confirms a moderate-to-large pooled effect for this narrowed projection (Amadeo et al., 2024; Casadio et al., 2024). Together, these findings suggest that schizophrenia involves impaired event construction in both past and future contexts and a narrowed temporal scope, limiting the autobiographical reference points necessary for accurate self-reflection and insight.

#### Temporal horizon and valuation

Temporal horizon refers to the span of time individuals can imagine and use to guide decisions, with a broader horizon supporting better anticipation of future outcomes and greater willingness to wait for delayed outcomes (Jones, Landes, Yi, & Bickel, 2009). In schizophrenia, both experimental and clinical studies indicate a ‘foreshortened future’ – difficulty extending prospection beyond the near term – and steeper delay discounting (greater preference for immediate rewards) (Jones et al., 2009). These alterations co-occur with documented deficits in episodic memory and future simulation, processes that support prospection and valuation of delayed outcomes (Chen et al., 2016).

Outside psychosis, episodic future thinking (EFT) – actively imagining specific future scenarios – reliably reduces delay discounting, increasing the value placed on delayed outcomes; meta-analyses show a robust overall effect, with positively valenced future cues producing the largest reductions (Rung & Madden, 2018; Ye et al., 2022). Although psychosis-specific EFT trials are scarce, schizophrenia shows consistent deficits in autobiographical/episodic memory and future simulation, processes that support prospection (Chen et al., 2016; Heerey, Matveeva, & Gold, 2011). Temporal-processing abnormalities are also documented in psychosis (e.g., widened audiovisual temporal-binding windows), underscoring a broader timing disturbance (Amadeo et al., 2024; Stevenson et al., 2017). A shortened temporal horizon compresses the window over which costs and benefits are evaluated, amplifying delay discounting and biasing choices toward immediate relief over delayed benefit. In schizophrenia, this specifically weakens the ‘need for treatment’ component of clinical insight, because future gains from medication or therapy are devalued relative to present inconvenience or side-effects. Together, these findings support a mechanistic bridge: expanding future simulation could improve appraisal of delayed treatment benefits – a computation relevant to the ‘need for treatment’ dimension of clinical insight and correction of poor illness awareness.

### Metacognition: the integrative hub

Metacognition refers to the ability to reflect on, monitor, and flexibly manage one’s own and others’ mental states. In schizophrenia, broad metacognitive impairments – especially in *self-reflectivity* (understanding one’s own thoughts) and *mastery* (using that understanding to respond adaptively) – are now considered core features of the disorder (Flavell, 1979). These deficits predict poorer clinical and cognitive insight, independent of neurocognitive performance or symptom severity, and have been shown to partially mediate the relationship between overgeneral autobiographical memory and reduced illness awareness (Davies & Greenwood, 2020; Mediavilla et al., 2021). In this framework, cognitive insight (BCIS) is treated as a metacognitive appraisal subdomain (self-reflection/self-certainty), whereas metacognition additionally includes mastery and related capacities not captured by BCIS, helping to explain distinct patterns of association with clinical insight.

Neuroimaging studies reveal that metacognitive self-reflection recruits the medial prefrontal cortex, precuneus, and posterior cingulate – key nodes within the default-mode network (DMN), a system in the brain that becomes active during rest, self-focused thinking, remembering the past, and imagining the future (Buckner, Andrews-Hanna, & Schacter, 2008). Disruptions in this network are common in schizophrenia and may explain overlapping problems in metacognition, autobiographical memory, and mental time travel. These same regions also support MTT and show functional disruption in patients with poor insight (Fuentes-Claramonte et al., 2019; Holt et al., 2011; Shan et al., 2020). Thus, metacognition may act as a central mechanism through which impoverished mental time travel simulations (due to degraded autobiographical memory) fail to transform into accurate, treatment-relevant insight.

## Behavioral evidence: how mental-time-travel performance tracks with insight

### Cross-sectional findings in established illness

Since 2005, around two dozen studies have shown that poorer performance on mental time travel tasks – such as episodic past recall (e.g., Autobiographical Interview), future-event simulation (e.g., Scene Construction, Future-Event Fluency), and temporal-horizon tasks – is consistently linked to lower insight in individuals with schizophrenia. After controlling for factors like IQ and negative symptoms, correlations between MTT measures and insight typically fall in the small-to-moderate range (*r* ≈ 0.30–0.45) (Barry, Hallford, Del Rey, & Ricarte, 2020; Berna et al., 2016; MacDougall et al., 2015; Raffard et al., 2010). This relationship holds across diverse samples, including outpatient and forensic populations, and is consistent across multiple measures of insight (e.g., SUMD, SAI-E, BCIS), regardless of whether patients primarily show positive or negative symptoms. When both components were analyzed, associations between MTT measures and clinical insight were generally stronger and more consistent than those with cognitive insight, although higher Self-Reflectiveness frequently accompanied greater autobiographical/future specificity.

Together, these findings suggest that MTT impairments are not just secondary effects or side issues – they are closely tied to the core clinical problem of poor illness awareness.

### Early-course and high-risk cohorts

Emerging evidence suggests that the link between MTT and insight is present early in the course of illness and even in individuals at elevated risk. In a study of first-episode patients, Potheegadoo et al. found that distorted perceptions of how close or distant past events felt – along with reduced episodic detail – explained about 11% of the variance in SUMD insight scores (*r* = .31) (Potheegadoo, Cuervo-Lombard, Berna, & Danion, 2012). Among high-schizotypy undergraduates, Hazan et al. reported that vague, goal-sparse future narratives and low perceived control in turning-point memories predicted weaker perceived need for help (*r* = −.28) on the Insight and Treatment Attitudes Questionnaire – ITAQ (Hazan, Reese, & Linscott, 2019).

In an early-course clinical sample, Allé et al. found that fragmented narratives across past and future life stories were linked to higher unawareness of illness (*β* = −.33) (Allé et al., 2016). Similarly, Barry et al. showed that in patients within 2 years of diagnosis, generating more specific future events predicted higher BCIS Self-Reflectiveness (*r* = .39) and lower Self-Certainty (*r* = −.35) (Barry et al., 2020). Even in non-clinical populations, less vivid and less detailed ‘delusion-like’ memories predicted stronger conviction in those thoughts (*β* = −.33) (Berna et al., 2016). Together, these findings suggest that impoverished scene construction, narrowed time horizons, and disrupted narrative continuity are not late effects of chronic schizophrenia, but early cognitive vulnerabilities that impair the development of accurate insight.

### Mediation and moderation analyses

#### Indirect evidence for metacognitive mediation

Although no published study has yet modelled the full chain ‘MTT detail → metacognition → clinical insight,’ converging evidence supports each link in the pathway. First, reduced autobiographical or episodic-memory specificity is consistently associated with weaker metacognitive-self capacities (Mediavilla et al., 2021), Second, lower metacognitive scores, in turn, predict poorer clinical insight (Lungu et al., 2023; Martiadis et al., 2023). For example, in individuals with attenuated psychotic symptoms, Berna et al. (2016) found that fewer autobiographical-memory details correlated with higher Self-Disorder scores – a construct that substantially overlaps the *Self-Reflectivity* dimension of the MAS-A (Berna et al., 2016). Similarly, studies in first-episode psychosis show that impaired metacognitive mastery is linked to diminished insight, whereas higher mastery scores independently predict better illness awareness (Leonhardt et al., 2020; Vohs et al., 2015). Taken together, these findings strongly suggest a mediation pathway, but a definitive test that incorporates a genuine MTT task (e.g., Scene Construction, TEMT) alongside standardized metacognition and insight measures remains an important research priority.

#### Symptom moderators

Emerging evidence suggests that the association between autobiographical memory (AM) specificity and clinical insight may be particularly pronounced in individuals with prominent negative symptoms. Meta-analyses and large-scale reviews consistently show that people with schizophrenia recall fewer specific autobiographical memories than healthy controls, with moderate-to-large effect sizes for memory specificity and richness of detail (Berna, Potheegadoo, et al., 2016; H. Zhang et al., 2019; Y. Zhang et al., 2019). Recent studies further indicate that AM performance is significantly correlated with negative symptoms, including apathy, in chronic schizophrenia. Specifically, one investigation found that the extent of negative symptoms may explain a substantial portion of AM deficits, with AM specificity and vividness both reduced in patients exhibiting higher negative symptom severity (Herold et al., 2023). Although most research confirms a general link between diminished AM specificity and poorer insight, these findings imply that in negative-symptom-dominant subgroups, deficits in autobiographical memory may have a more substantial impact on self-awareness and illness recognition, thereby amplifying the coupling between memory specificity and clinical insight (Herold et al., 2023). This pattern underscores the importance of considering symptom profiles when examining cognitive mechanisms underlying insight in schizophrenia.

#### Affective and trauma moderators

Mood and life-history factors appear to shape how strongly autobiographical-memory deficits translate into poor insight. Several studies show that higher depressive symptoms are paradoxically associated with better illness awareness (Lincoln et al., 2007; Lysaker et al., 2018; Weiss-Cowie, Verhaeghen, & Duarte, 2023), a pattern often interpreted as ‘depressive realism’ (Moore & Fresco, 2012). By contrast, childhood-trauma exposure has been linked both to over-general autobiographical memory and to diminished clinical insight (Berenz et al., 2018; Irwin, Green, & Marsh, 1999; Kalantar-Hormozi & Mohammadkhani, 2024). More recent work in serious mental-illness samples indicates that PTSD symptom severity co-occurs with fragmented personal memories (Hardy, 2017). Although no published study has yet tested these variables as formal moderators of an MTT and insight pathway, the converging evidence suggests that dysphoric mood may *attenuate* – and trauma history may *amplify* – the cognitive route to impaired insight.

Higher depressive symptoms are often linked to greater clinical insight in schizophrenia (the ‘insight paradox’), plausibly via demoralization/internalized stigma and rumination-driven negative self-appraisal – pathways that raise illness acknowledgment independently of MTT; consequently, dysphoria can attenuate observed MTT–insight correlations (Belvederi Murri et al., 2015; Cavelti et al., 2012; Lysaker, Gagen, Moritz, & Schweitzer, 2018).

#### Interim synthesis – **strengths, limits, and open questions**

**Figure 1.**
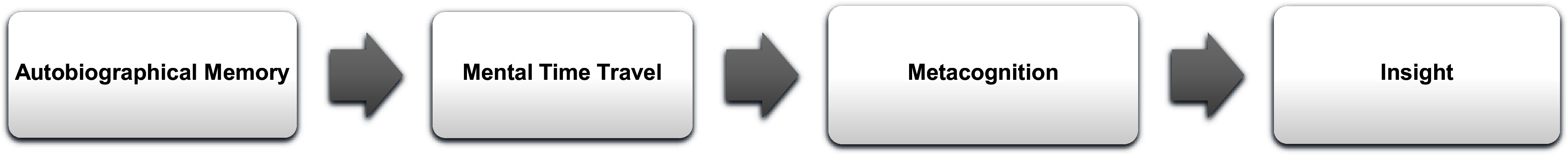
Conceptual pathway from autobiographical memory to clinical insight in schizophrenia.

Behavioral findings converge on a plausible chain – impoverished MTT performance → weaker metacognitive monitoring → poorer clinical insight – and show that delusional conviction, prominent negative symptoms, and trauma intensify this pathway, whereas depressive mood may blunt it. This proposed pathway is depicted in Figure 1. (Balzan et al., 2019; Faith et al., 2020; Luther et al., 2020; Vohs et al., 2014). Yet three key gaps remain: (i) MTT-specific mechanisms are still opaque. Most studies measure global metacognition; only a handful deploy dedicated MTT tasks (scene construction, temporal-horizon) when examining insight. Whether the *episodic* component uniquely drives insight loss is therefore unproven (Lysaker, Gagen, et al., 2018). (ii) Trauma’s role is only indirectly supported. Childhood adversity fragments autobiographical memory and weakens metacognition, but no published work has tested trauma × MTT or trauma × insight interactions in a single model (Aharon Biram et al., 2024; Takarangi, Smith, Strange, & Flowe, 2017). (iii) Causality is unknown. Evidence is almost entirely cross-sectional, so we cannot determine whether MTT deficits *cause* poor insight, whether poor insight erodes MTT, or whether both stem from a shared neural factor such as default-mode dysconnectivity – an idea awaiting longitudinal or interventional tests (Jun, Miao, & Ying, 2025). In sum, therapies that jointly enhance episodic specificity and metacognitive mastery (e.g., Metacognitive Reflection and Insight Therapy (MERIT), episodic-future-thinking modules) remain the best-supported clinical options (de Jong et al., 2019; Martin et al., 2023), but definitive studies – longitudinal, experimental, and trauma-stratified – are still needed to confirm the direction and specificity of the MTT → metacognition → insight pathway.

## Neuroimaging and neurophysiology

### Task-based fMRI: overlapping circuitry for MTT and insight

Core brain regions jointly implicated in MTT and insight—particularly the medial prefrontal cortex, posterior cingulate cortex, and hippocampus—are summarized in Table 1. Advanced neuroimaging studies increasingly support the idea that MTT and insight rely on overlapping brain networks (DMN) (Østby et al., 2012; Viard et al., 2011). Moving beyond traditional univariate contrasts, multivariate fMRI methods such as spatial independent component analysis (sICA) show that seemingly different tasks – like episodic recall, future simulation, and self-appraisal – activate common functional networks (Xu et al., 2015). These shared networks often involve the medial prefrontal cortex, posterior cingulate cortex, and hippocampus, where the same brain regions show synchronized activation patterns across task types (Dafni-Merom et al., 2024).

**Table 1.** Core brain regions shared by mental time travel (MTT) and insight in schizophrenia. These three regions – part of the default mode network – are consistently implicated in both autobiographical memory processes and self-reflective functions. Their disruption may underlie the co-occurring deficits in MTT and illness awareness observed in schizophrenia

Brain region	Role in MTT	Role in insight
Medial Prefrontal Cortex (mPFC)	Self-related thinking, future imagination	Judging one’s own mental state, illness awareness
Posterior Cingulate Cortex (PCC)	Autobiographical memory, scene recall	Self-reflection, awareness of internal states
Hippocampus	Memory specificity, scene construction	Provides episodic details for insight

Dynamic connectivity studies (‘connectotyping’) show that connections among fronto-hippocampal and default-mode regions reconfigure on the order of seconds as people move through different task stages, rather than remaining static (Miranda-Dominguez et al., 2014; Vazquez-Trejo et al., 2022). Time-resolved approaches capture brief, recurring interaction ‘states,’ highlighting flexible adaptation of network coupling to cognitive demands. This rapid reconfiguration is particularly relevant for fronto-hippocampal communication that supports self-referential processing, episodic retrieval, and prospection (Campbell et al., 2018; Molnar-Szakacs & Uddin, 2013). In schizophrenia, multiple studies report altered dynamic functional connectivity – reduced time in highly integrated states, longer dwell in weakly connected states, and fewer state transitions – patterns linked to symptom burden and cognitive dysfunction; such instability plausibly undermines metacognitive operations and clinical insight that depend on fluid shifts between internal mentation and external task focus (Lysaker et al., 2019; Sendi et al., 2021; Shan et al., 2020; Weber et al., 2020; You et al., 2022).

Other methods, such as non-negative matrix factorization (NMF), isolate partially overlapping brain networks and can support multivariate decoding of task states. These network patterns have even been used to classify what a person is doing – for example, constructing a mental scene, evaluating personal traits, or making a simple decision (Aggarwal & Gupta, 2018; Anderson et al., 2014; Shirer et al., 2012). Importantly, ventromedial prefrontal and frontoparietal regions often carry high weights in both MTT and insight-related tasks, suggesting that these domains rely on shared control/default-adjacent components (Aggarwal & Gupta, 2018; Anderson et al., 2014; Cole et al., 2013; Vincent et al., 2008). Here, ‘insight-related tasks’ refer to self-/other trait-judgment paradigms linked to SAI-E/BCIS, reality/source-monitoring with confidence reports, error-awareness paradigms (ACC-mediated), and probabilistic ‘jumping-to-conclusions’ tasks, each recruiting vmPFC and frontoparietal control circuitry (Andreou et al., 2018; Bedford et al., 2012; Carter, MacDonald III, Ross, & Stenger, 2001; Garrison, Fernandez-Egea, Zaman, Agius, & Simons, 2017; Hester, Nestor, & Garavan, 2009; Simons, Garrison, & Johnson, 2017; van der Meer et al., 2013). Large-scale analyses pooling data across many task paradigms (8 or more) consistently point to the frontoparietal control network as a key integrative hub. Activity in this network predicts performance in tasks requiring episodic future thinking, autobiographical memory, and clinical insight (Albouy, Martinez-Moreno, Hoyer, Zatorre, & Baillet, 2022; Pagnotta, Riddle, & D’Esposito, 2024; Vanasse et al., 2021). Some researchers note that traditional GLM-based fMRI analyses may miss this overlap, because they average out rapid shifts in connectivity that multivariate methods are better equipped to detect (Fang, Poskanzer, & Anzellotti, 2023; Moeller & Habeck, 2006; Salvador et al., 2020). Taken together, these findings support the idea that MTT and insight do not rely on separate, isolated brain systems. Instead, they draw on a partially overlapping and dynamically coordinated neural architecture (Brocas & Carrillo, 2018; Dafni-Merom et al., 2024; Gauthier & van Wassenhove, 2016).

### Resting-state connectivity: trait-level links between network integrity, MTT, and insight

Schizophrenia is reliably associated with altered connectivity within the DMN, most notably reduced (hypo-)connectivity between the vmPFC and the PCC (Dong et al., 2018; Peng et al., 2021), as well as between the PCC and the hippocampus (Dugré et al., 2021). Meta-analyses estimate these disruptions to be moderate in magnitude (Cohen’s *d* ≈ 0.4–0.6). At the same time, early or prodromal findings are heterogeneous: while many reports emphasize hypoconnectivity, some studies in CHR/early-course samples describe increased vmPFC–PCC connectivity (hyperconnectivity), particularly among individuals with poorer clinical insight (Clark et al., 2018; O’Neill, Mechelli, & Bhattacharyya, 2019), whereas other early-onset cohorts show reductions (e.g., Hilland et al., 2022) Accordingly, the literature search was broadened to include clinical high-risk (CHR)/ultra-high-risk (UHR) and first-episode cohorts; stage and analytic choices (e.g., global signal regression, parcellation, motion handling) likely moderate the direction of observed DMN effects. These DMN connectivity abnormalities are thought to underlie impairments in self-referential processing, autobiographical memory, and metacognition observed in schizophrenia.

Crucially, the weaker these connections, the fewer episodic details patients recall during the *Autobiographical Interview*, and the lower their clinical insight, as measured by SUMD scores (Fan et al., 2022). Connectivity between the anterior hippocampus and vmPFC is especially important: reduced coupling in this pathway predicts both less vivid future-event construction and lower self-reflectiveness on the BCIS.

In addition, graph-theory studies show reduced global efficiency and weaker hub integrity across the DMN and frontoparietal control networks. These network-level disruptions explain unique variance in SAI-E insight scores – even after accounting for symptom severity (Blessing et al., 2020; Du et al., 2016; Dugré et al., 2021; Livingston et al., 2024; Micheloyannis, 2012; Pan et al., 2022).

Dynamic connectivity analyses add a time-based perspective. Patients spend less time in a brain state dominated by DMN and control-network activity, and more time in a sensorimotor-dominated state. Notably, shorter ‘high-DMN’ dwell time predicts both poorer future-event fluency and lower insight 6 months later (Du et al., 2016; Fox et al., 2017; Sendi et al., 2021).

Together, these resting-state findings mirror task-based deficits. They suggest that predominantly weakened connectivity among the vmPFC, precuneus, and hippocampus, while accommodating early-stage reports of hyperconnectivity, disrupts the flow of episodic content into metacognitive systems, ultimately degrading clinical insight (Fornara et al., 2017; Gee et al., 2025; Kühn & Gallinat, 2013).

### Electrophysiology: millisecond-scale evidence for a predictive-processing link

EEG studies show that insight failures in schizophrenia begin within milliseconds of processing information. Reduced error-related negativity (ERN) in the medial frontal cortex and lower mismatch negativity (MMN) – both markers of disrupted predictive coding – are linked to fewer episodic memory details and poorer clinical insight (higher SUMD scores) (Hamilton, Boos, & Mathalon, 2020; Kansal, Patriciu, & Kiang, 2014; Perrottelli et al., 2022). Additionally, a smaller P300 response to self-relevant cues is associated with lower self-reflectiveness on the BCIS. Together, these findings suggest that weak early brain signals related to prediction errors and salience detection deprive metacognitive systems of the moment-by-moment input needed for vivid mental scene construction – ultimately contributing to impaired insight (Lysaker et al., 2019; Lysaker, Pattison, et al., 2018). Electrophysiological findings suggest that fast, early brain signals involved in prediction and self-relevance processing are weakened in schizophrenia, contributing to poor memory detail and impaired insight. Importantly, EEG/magnetoencephalography (MEG) studies of mental time travel show that constructing past and future events elicits a parietal late positive component and late frontal monitoring effects, and engages hippocampal–vmPFC theta interactions; these millisecond-scale signatures track episodic detail and temporal distance (Barry, Barnes, Clark, & Maguire, 2019; Colás-Blanco, Mioche, La Corte, & Piolino, 2022; Monk, Barnes, & Maguire, 2020).

### Multimodal & molecular imaging: converging structural evidence

Findings from structural MRI, diffusion tensor imaging (DTI), and PET all support the same hippocampal–vmPFC circuit implicated in fMRI and EEG studies. Smaller volumes in the anterior hippocampus and ventromedial prefrontal cortex (vmPFC) are linked to both fewer autobiographical memory details and poorer insight (effect sizes *d* ≈ 0.4–0.5) (Adriano, Caltagirone, & Spalletta, 2012; Duan et al., 2020; Dugré et al., 2021). In addition, reduced fractional anisotropy in the uncinate fasciculus – the main white-matter tract connecting the anterior hippocampus and vmPFC – has been shown to partially mediate the relationship between mental time travel abilities and clinical insight (Herold et al., 2013; Kelly et al., 2018; Lysaker & Dimaggio, 2014; Samartzis, Dima, Fusar-Poli, & Kyriakopoulos, 2014; Von Der Heide, Skipper, Klobusicky, & Olson, 2013). This supports a structural basis for failures in integrating episodic memory content into metacognitive awareness.

Taken together, these findings suggest that a structurally and chemically weakened hippocampal–vmPFC loop acts as a ‘hardware bottleneck’ through which impoverished MTT content reaches the systems responsible for self-reflection and insight (Lysaker & Dimaggio, 2014; McCormick, Ciaramelli, De Luca, & Maguire, 2018). However, longitudinal multimodal studies that incorporate explicit MTT tasks are still needed to confirm the direction and specificity of this circuit’s role in insight.

## Mechanistic & translational implications

A predictive-processing framework best explains the converging evidence: a structurally and neurochemically weakened hippocampal–vmPFC circuit produces low-precision episodic priors, weakens early prediction-error signals (MMN/ERN), and deprives metacognitive systems of the rich autobiographical input needed for self-evaluation – leading to poor clinical insight (Erickson, Ruffle, & Gold, 2016; Kelly et al., 2018; McCormick et al., 2018). PET studies suggest that reduced dopamine synthesis in the vmPFC and excess glutamate in the hippocampus may further reduce the precision of these priors, reinforcing delusional conviction and negative-symptom inertia (Egerton, Modinos, Ferrera, & McGuire, 2017; Slifstein et al., 2015). Translationally, two intervention levers emerge: (i) sharpen episodic specificity via episodic-future-thinking drills, memory-specificity training, or VR ‘future-self’ modules; (ii) enhance metacognitive mastery through Metacognitive Reflection and Insight Therapy (MERIT) or insight-focused CBT add-ons (Hasson-Ohayon et al., 2024; Ye et al., 2022). Pilot RCTs combining these components yield medium effect-size improvements on BCIS and SAI-E, with the strongest gains in highly delusional or trauma-exposed subgroups (López-Morínigo et al., 2023). Future trials should stratify by trauma and symptom profile, incorporate pre-/post-MTT tasks plus vmPFC–hippocampal rs-fMRI, and test precision-boosting pharmacological adjuncts (e.g., low-dose d-cycloserine, vortioxetine) (Diminich et al., 2020; Redaelli et al., 2022).

## Clinical implications and future directions

This model has important implications for both clinical practice and future research. By highlighting the role of impaired MTT and metacognition in the emergence of poor insight, it reframes insight not merely as a fixed symptom but as a dynamic cognitive process that may be amenable to intervention. Clinically, early screening for deficits in autobiographical memory, future simulation, or self-reflective reasoning could help identify patients at risk for persistent insight impairment – particularly in early-course or high-risk populations.

Integrating both cognitive and neural measures – such as scene-construction tasks, BCIS scores, and vmPFC–hippocampal connectivity – may enable more personalized treatment planning. Future research should prioritize longitudinal, stratified trials that test combined cognitive-metacognitive interventions, monitor insight changes over time, and evaluate the utility of MTT-based paradigms as prognostic biomarkers for insight recovery. Ultimately, targeting the MTT–metacognition–insight pathway may enhance engagement, adherence, and long-term functional outcomes in individuals with schizophrenia.

## Limitations and conclusion

While this review offers a unified model linking MTT, metacognition, and clinical insight in schizophrenia, several limitations should be noted. Importantly, almost all existing evidence is cross-sectional in nature, making it difficult to determine causal relationships. It remains unclear whether impaired MTT leads to poor insight, whether the reverse is true, or whether both stem from shared disruptions in neural systems like the default mode network. Reverse causality and bidirectional effects are plausible and represent important open questions.

Additionally, the wide variation in how MTT and insight are measured – across both behavioral and neuroimaging studies – limits direct comparisons and meta-analytic synthesis. Insight is often assessed using clinician ratings, which may not fully capture subjective, fluctuating, or motivational aspects of self-awareness.

Moreover, few studies have tested full mediation models that simultaneously assess MTT, metacognition, and insight in the same population, limiting mechanistic understanding. Important moderators such as trauma exposure, symptom subtype, and medication effects are often underreported, despite evidence that they shape both cognitive and neural outcomes. Finally, although structural, functional, and molecular imaging findings broadly converge, truly integrated multimodal studies that link these domains in individual patients remain scarce.

Despite these gaps, the available data support a promising mechanistic framework: that impoverished MTT appears to limit the episodic content available to metacognitive systems, weakening self-reflection and ultimately degrading insight. This pathway is supported by converging evidence from fMRI, EEG, structural imaging, and behavioral tasks. Clinically, targeting both episodic specificity and metacognitive mastery – through cognitive drills, metacognitive therapy, and precision-enhancing pharmacological agents – may improve insight and long-term outcomes in schizophrenia. However, prospective longitudinal and stratified trials remain critically needed to validate this model, clarify causality, and establish whether MTT-based measures can serve as cognitive biomarkers or therapeutic targets for insight recovery.

## References

[r1] Adriano, F., Caltagirone, C., & Spalletta, G. (2012). Hippocampal volume reduction in first-episode and chronic schizophrenia: A review and meta-analysis. The Neuroscientist: A Review Journal Bringing Neurobiology, Neurology and Psychiatry, 18(2), 180–200. 10.1177/1073858410395147. 21531988

[r2] Aggarwal, P., & Gupta, A. (2018). Low rank and sparsity constrained method for identifying overlapping functional brain networks. PLoS One, 13(11), e0208068. 10.1371/journal.pone.0208068. 30485369 PMC6261626

[r3] Aharon Biram, S., Horesh, D., Tuval-Mashiach, R., & Hasson-Ohayon, I. (2024). World assumptions and post-traumatic symptoms: The moderating role of metacognition. European Journal of Trauma & Dissociation, 8(1), 100389. 10.1016/j.ejtd.2024.100389.

[r4] Albouy, P., Martinez-Moreno, Z. E., Hoyer, R. S., Zatorre, R. J., & Baillet, S. (2022). Supramodality of neural entrainment: Rhythmic visual stimulation causally enhances auditory working memory performance. Science Advances, 8(8), eabj9782. 10.1126/sciadv.abj9782. 35196074 PMC8865801

[r5] Allé, M. C., d’Argembeau, A., Schneider, P., Potheegadoo, J., Coutelle, R., Danion, J.-M., & Berna, F. (2016). Self-continuity across time in schizophrenia: An exploration of phenomenological and narrative continuity in the past and future. Comprehensive Psychiatry, 69, 53–61. 10.1016/j.comppsych.2016.05.001. 27423345

[r6] Amadeo, M. B., Escelsior, A., Esposito, D., Inuggi, A., Versaggi, S., Marenco, G., … Gori, M. (2024). Multisensory temporal processing in schizophrenia and bipolar disorder: Implications for psychosis. Schizophrenia, 10, 98. 10.1038/s41537-024-00502-z. 39477981 PMC11526139

[r7] Amador, X. F., & Kronengold, H. (2004). Understanding and assessing insight. In X. F. Amador & A. S. David (Eds.), Insight and psychosis: Awareness of illness in schizophrenia and related disorders (2nd ed., pp. 3–30). Oxford University Press. 10.1093/med/9780198525684.003.0001

[r8] Anderson, A., Douglas, P. K., Kerr, W. T., Haynes, V. S., Yuille, A. L., Xie, J., **…** Cohen, M. S. (2014). Non-negative matrix factorization of multimodal MRI, fMRI, and phenotypic data reveals differential changes in default mode subnetworks in ADHD. NeuroImage, 102(Pt 1), 207–219. 10.1016/j.neuroimage.2013.12.015. 24361664 PMC4063903

[r9] Andreou, C., Steinmann, S., Leicht, G., Kolbeck, K., Moritz, S., & Mulert, C. (2018). fMRI correlates of jumping-to-conclusions in patients with delusions: Connectivity patterns and effects of metacognitive training. NeuroImage. Clinical, 20, 119–127. 10.1016/j.nicl.2018.07.004. 30094162 PMC6077165

[r10] Balzan, R. P., Mattiske, J. K., Delfabbro, P., Liu, D., & Galletly, C. (2019). Individualized metacognitive training (MCT+) reduces delusional symptoms in psychosis: A randomized clinical trial. Schizophrenia Bulletin, 45(1), 27–36. 10.1093/schbul/sby152. 30376124 PMC6293215

[r11] Barry, D. N., Barnes, G. R., Clark, I. A., & Maguire, E. A. (2019). The neural dynamics of novel scene imagery. The Journal of neuroscience: the official journal of the Society for Neuroscience, 39(22), 4375–4386. 10.1523/JNEUROSCI.2497-18.2019. 30902867 PMC6538850

[r12] Barry, T. J., Hallford, D. J., Del Rey, F., & Ricarte, J. J. (2020). Differential associations between impaired autobiographical memory recall and future thinking in people with and without schizophrenia. The British Journal of Clinical Psychology, 59(2), 154–168. 10.1111/bjc.12236. 31584204

[r13] Beck, A. T., Baruch, E., Balter, J. M., Steer, R. A., & Warman, D. M. (2004). A new instrument for measuring insight: The Beck cognitive insight scale. Schizophrenia Research, 68(2–3), 319–329. 10.1016/S0920-9964(03)00189-0. 15099613

[r14] Bedford, N. J., Surguladze, S., Giampietro, V., Brammer, M. J., & David, A. S. (2012). Self-evaluation in schizophrenia: An fMRI study with implications for the understanding of insight. BMC Psychiatry, 12, 106. 10.1186/1471-244X-12-106. 22876974 PMC3527271

[r15] Belvederi Murri, M., Respino, M., Innamorati, M., Cervetti, A., Calcagno, P., Pompili, M., … Amore, M. (2015). Is good insight associated with depression among patients with schizophrenia? Systematic review and meta-analysis. Schizophrenia Research, 162(1–3), 234–247. 10.1016/j.schres.2015.01.003. 25631453

[r16] Berenz, E. C., Vujanovic, A., Rappaport, L. M., Kevorkian, S., Gonzalez, R. E., Chowdhury, N., Dutcher, C., Dick, D. M., Kendler, K. S., & Amstadter, A. (2018). A multimodal study of childhood trauma and distress tolerance in young adulthood. Journal of Aggression, Maltreatment & Trauma, 27(7), 795–810. 10.1080/10926771.2017.1382636. PMC6329603 30636862

[r17] Berna, F., Göritz, A. S., Schröder, J., Martin, B., Cermolacce, M., Allé, M. C., Danion, J. M., Cuervo-Lombard, C. V., & Moritz, S. (2016). Self-disorders in individuals with attenuated psychotic symptoms: Contribution of a dysfunction of autobiographical memory. Psychiatry Research, 239, 333–341. 10.1016/j.psychres.2016.03.029. 27058160

[r18] Berna, F., Potheegadoo, J., Aouadi, I., Ricarte, J. J., Allé, M. C., Coutelle, R., Boyer, L., Cuervo-Lombard, C. V., & Danion, J.-M. (2016). A meta-analysis of autobiographical memory studies in schizophrenia Spectrum disorder. Schizophrenia Bulletin, 42(1), 56–66. 10.1093/schbul/sbv099. 26209548 PMC4681554

[r19] Blessing, E. M., Murty, V. P., Zeng, B., Wang, J., Davachi, L., & Goff, D. C. (2020). Anterior hippocampal–cortical functional connectivity distinguishes antipsychotic naïve first-episode psychosis patients from controls and may predict response to second-generation antipsychotic treatment. Schizophrenia Bulletin, 46(3), 680–689. 10.1093/schbul/sbz076. 31433843 PMC7147586

[r20] Bréchet, L. (2022). Personal memories and bodily-cues influence our sense of self. Frontiers in Psychology, 13. 10.3389/fpsyg.2022.855450. PMC9257125 35814046

[r21] Brocas, I., & Carrillo, J. D. (2018). A Neuroeconomic theory of mental time travel. Frontiers in Neuroscience, 12. 10.3389/fnins.2018.00658. PMC6168654 30319339

[r22] Bröcker, A. L., Bayer, S., Stuke, F., Giemsa, P., Heinz, A., Bermpohl, F., … Montag, C. (2017). The metacognition assessment scale (MAS-A): Results of a pilot study applying a German translation to individuals with schizophrenia spectrum disorders. Psychology and Psychotherapy, 90(3), 401–418. 10.1111/papt.12122. 28334488

[r23] Buckner, R. L., Andrews-Hanna, J. R., & Schacter, D. L. (2008). The brain’s default network: Anatomy, function, and relevance to disease. Annals of the New York Academy of Sciences, 1124, 1–38. 10.1196/annals.1440.011. 18400922

[r24] Campbell, K. L., Madore, K. P., Benoit, R. G., Thakral, P. P., & Schacter, D. L. (2018). Increased hippocampus to ventromedial prefrontal connectivity during the construction of episodic future events. Hippocampus, 28(2), 76–80. 10.1002/hipo.22812. 29116660 PMC5777865

[r25] Carter, C. S., MacDonald III, A. W., Ross, L. L., & Stenger, V. A. (2001). Anterior cingulate cortex activity and impaired self-monitoring of performance in patients with schizophrenia: An event-related fMRI study. The American Journal of Psychiatry, 158(9), 1423–1428. 10.1176/appi.ajp.158.9.1423 11532726

[r26] Casadio, C., Patané, I., Candini, M., Lui, F., Frassinetti, F., & Benuzzi, F. (2024). Effects of the perceived temporal distance of events on mental time travel and on its underlying brain circuits. Experimental Brain Research, 242(5), 1161–1174. 10.1007/s00221-024-06806-x. 38489024 PMC11078804

[r27] Cavelti, M., Kvrgic, S., Beck, E. M., Rüsch, N., & Vauth, R. (2012). Self-stigma and its relationship with insight, demoralization, and clinical outcome among people with schizophrenia spectrum disorders. Comprehensive Psychiatry, 53(5), 468–479. 10.1016/j.comppsych.2011.08.001. 21956043

[r28] Chen, X. J., Liu, L. L., Cui, J. F., Wang, Y., Chen, A. T., Li, F. H., … Chan, R. C. (2016). Schizophrenia Spectrum disorders show reduced specificity and less positive events in mental time travel. Frontiers in Psychology, 7, 1121. 10.3389/fpsyg.2016.01121. 27507958 PMC4960265

[r29] Clark, S. V., Mittal, V. A., Bernard, J. A., Ahmadi, A., King, T. Z., & Turner, J. A. (2018). Stronger default mode network connectivity is associated with poorer clinical insight in youth at ultra high-risk for psychotic disorders. Schizophrenia Research, 193, 244–250. 10.1016/j.schres.2017.06.043. 28688741 PMC5756141

[r30] Colás-Blanco, I., Mioche, J., La Corte, V., & Piolino, P. (2022). The role of temporal distance of the events on the spatiotemporal dynamics of mental time travel to one’s personal past and future. Scientific Reports, 12(1), 2378. 10.1038/s41598-022-05902-8. 35149740 PMC8837801

[r31] Cole, M. W., Reynolds, J. R., Power, J. D., Repovs, G., Anticevic, A., & Braver, T. S. (2013). Multi-task connectivity reveals flexible hubs for adaptive task control. Nature Neuroscience, 16(9), 1348–1355. 10.1038/nn.3470. 23892552 PMC3758404

[r32] Cooke, M. A., Peters, E. R., Fannon, D., Aasen, I., Kuipers, E., & Kumari, V. (2010). Cognitive insight in psychosis: The relationship between self-certainty and self-reflection dimensions and neuropsychological measures. Psychiatry Research, 178(2), 284–289. 10.1016/j.psychres.2009.05.009. 20483170 PMC3184477

[r33] Dafni-Merom, A., Monsa, R., Benbaji, M., Klein, A., & Arzy, S. (2024). Travelling beyond time: Shared brain system for self-projection in the temporal, political and moral domains. Philosophical Transactions of the Royal Society of London. Series B, Biological Sciences, 379(1913), rstb20230414. 10.1098/rstb.2023.0414. 39278258 PMC11449160

[r34] Danion, J.-M., Huron, C., Vidailhet, P., & Berna, F. (2007). Functional mechanisms of episodic memory impairment in schizophrenia. Canadian Journal of Psychiatry. Revue Canadienne de Psychiatrie, 52(11), 693–701. 10.1177/070674370705201103. 18399036

[r35] David, A. S. (1990). Insight and psychosis. The British Journal of Psychiatry: the Journal of Mental Science, 156, 798–808. 10.1192/bjp.156.6.798. 2207510

[r36] David, A., Buchanan, A., Reed, A., & Almeida, O. (1992). The assessment of insight in psychosis. The British Journal of Psychiatry: the Journal of Mental Science, 161, 599–602. 10.1192/bjp.161.5.599. 1422606

[r37] Davies, G., & Greenwood, K. (2020). A meta-analytic review of the relationship between neurocognition, metacognition and functional outcome in schizophrenia. Journal of Mental Health (Abingdon, England), 29(5), 496–505. 10.1080/09638237.2018.1521930. 30378436

[r38] de Jong, S., van Donkersgoed, R. J. M., Timmerman, M. E., Aan Het Rot, M., Wunderink, L., Arends, J., van Der Gaag, M., Aleman, A., Lysaker, P. H., & Pijnenborg, G. H. M. (2019). Metacognitive reflection and insight therapy (MERIT) for patients with schizophrenia. Psychological Medicine, 49(2), 303–313. 10.1017/S0033291718000855. 29692285

[r39] Diminich, E. D., Dickerson, F., Bello, I., Cather, C., Kingdon, D., Rakhshan Rouhakhtar, P. J., … Goff, D. C. (2020). D-cycloserine augmentation of cognitive behavioral therapy for delusions: A randomized clinical trial. Schizophrenia Research, 222, 145–152. 10.1016/j.schres.2020.06.015. 32591238

[r40] Dong, D., Wang, Y., Chang, X., Luo, C., & Yao, D. (2018). Dysfunction of large-scale brain networks in schizophrenia: A meta-analysis of resting-state functional connectivity. Schizophrenia Bulletin, 44(1), 168–181. 10.1093/schbul/sbx034. 28338943 PMC5767956

[r41] Du, Y., Pearlson, G. D., Yu, Q., He, H., Lin, D., Sui, J., Wu, L., & Calhoun, V. D. (2016). Interaction among subsystems within default mode network diminished in schizophrenia patients: A dynamic connectivity approach. Schizophrenia Research, 170(1), 55–65. 10.1016/j.schres.2015.11.021. 26654933 PMC4707124

[r42] Duan, X., He, C., Ou, J., Wang, R., Xiao, J., Li, L., … Chen, H. (2020). Reduced hippocampal volume and its relationship with verbal memory and negative symptoms in treatment-naive first-episode adolescent-onset schizophrenia. Schizophrenia Bulletin, 47(1), 64–74. 10.1093/schbul/sbaa092. PMC7825026 32691057

[r43] Dugré, J. R., Dumais, A., Tikasz, A., Mendrek, A., & Potvin, S. (2021). Functional connectivity abnormalities of the long-axis hippocampal subregions in schizophrenia during episodic memory. NPJ Schizophrenia, 7, 19. 10.1038/s41537-021-00147-2. 33658524 PMC7930183

[r44] Egerton, A., Modinos, G., Ferrera, D., & McGuire, P. (2017). Neuroimaging studies of GABA in schizophrenia: A systematic review with meta-analysis. Translational Psychiatry, 7(6), e1147. 10.1038/tp.2017.124. 28585933 PMC5537645

[r45] Engh, J. A., Friis, S., Birkenaes, A. B., Jónsdóttir, H., Ringen, P. A., Ruud, T., … Andreassen, O. A. (2007). Measuring cognitive insight in schizophrenia and bipolar disorder: A comparative study. BMC Psychiatry, 7, 71. 10.1186/1471-244X-7-71. 18072961 PMC2222246

[r46] Erickson, M. A., Ruffle, A., & Gold, J. M. (2016). A meta-analysis of mismatch negativity in schizophrenia: From clinical risk to disease specificity and progression. Biological Psychiatry, 79(12), 980–987. 10.1016/j.biopsych.2015.08.025. 26444073 PMC4775447

[r47] Faith, L. A., Lecomte, T., Corbière, M., Francoeur, A., Hache-Labelle, C., & Lysaker, P. H. (2020). Metacognition is uniquely related to concurrent and prospective assessments of negative symptoms independent of verbal memory in serious mental illness. The Journal of Nervous and Mental Disease, 208(11), 837. 10.1097/NMD.0000000000001219. 32740145

[r48] Fan, F., Tan, S., Huang, J., Chen, S., Fan, H., Wang, Z., … Tan, Y. (2022). Functional disconnection between subsystems of the default mode network in schizophrenia. Psychological Medicine, 52(12), 2270–2280. 10.1017/S003329172000416X. 33183375

[r49] Fang, M., Poskanzer, C., & Anzellotti, S. (2023). Multivariate connectivity: A brief introduction and an open question. Frontiers in Neuroscience, 16. 10.3389/fnins.2022.1082120. PMC9871770 36704006

[r50] Fivush, R. (2011). The development of autobiographical memory. Annual Review of Psychology, 62, 559–582. 10.1146/annurev.psych.121208.131702. 20636128

[r51] Flavell, J. H. (1979). Metacognition and cognitive monitoring: A new area of cognitive–developmental inquiry. American Psychologist, 34(10), 906–911. 10.1037/0003-066X.34.10.906.

[r52] Fornara, G. A., Papagno, C., & Berlingeri, M. (2017). A neuroanatomical account of mental time travelling in schizophrenia: A meta-analysis of functional and structural neuroimaging data. Neuroscience & Biobehavioral Reviews, 80, 211–222. 10.1016/j.neubiorev.2017.05.027. 28576509

[r53] Fox, J. M., Abram, S. V., Reilly, J. L., Eack, S., Goldman, M. B., Csernansky, J. G., … Smith, M. J. (2017). Default mode functional connectivity is associated with social functioning in schizophrenia. Journal of Abnormal Psychology, 126(4), 392–405. 10.1037/abn0000253. 28358526 PMC5418083

[r54] Fuentes-Claramonte, P., Martin-Subero, M., Salgado-Pineda, P., Santo-Angles, A., Argila-Plaza, I., Salavert, J., … Salvador, R. (2019). Brain imaging correlates of self- and other-reflection in schizophrenia. NeuroImage: Clinical, 25, 102134. 10.1016/j.nicl.2019.102134. 31877452 PMC6931228

[r55] Garrison, J. R., Fernandez-Egea, E., Zaman, R., Agius, M., & Simons, J. S. (2017). Reality monitoring impairment in schizophrenia reflects specific prefrontal cortex dysfunction. NeuroImage. Clinical, 14, 260–268. 10.1016/j.nicl.2017.01.028. 28203529 PMC5292760

[r56] Gauthier, B., & van Wassenhove, V. (2016). Time is not space: Core computations and domain-specific networks for mental travels. The Journal of Neuroscience, 36(47), 11891–11903. 10.1523/JNEUROSCI.1400-16.2016. 27881776 PMC6604919

[r57] Gee, A., Dazzan, P., Grace, A. A., & Modinos, G. (2025). Corticolimbic circuitry as a druggable target in schizophrenia spectrum disorders: A narrative review. Translational Psychiatry, 15(1), 21. 10.1038/s41398-024-03221-2. 39856031 PMC11760974

[r58] Hamilton, H. K., Boos, A., & Mathalon, D. H. (2020). Electroencephalography and event-related potential biomarkers in individuals at clinical high risk for psychosis. Biological Psychiatry, 88(4), 294–303. 10.1016/j.biopsych.2020.04.002. 32507388 PMC8300573

[r59] Hardy, A. (2017). Pathways from trauma to psychotic experiences: A theoretically informed model of posttraumatic stress in psychosis. Frontiers in Psychology, 8. 10.3389/fpsyg.2017.00697. PMC5440889 28588514

[r60] Hasson-Ohayon, I., Igra, L., Lavi-Rotenberg, A., Goldzweig, G., & Lysaker, P. H. (2024). Findings from a randomized controlled trial of metacognitive reflection and insight therapy for people with schizophrenia: Effects on metacognition and symptoms. Psychology and Psychotherapy, 97(Suppl 1), 75–90. 10.1111/papt.12485. 37522576

[r61] Hazan, H., Reese, E. J., & Linscott, R. J. (2019). Narrative self and high risk for schizophrenia: Remembering the past and imagining the future. Memory (Hove, England), 27(9), 1214–1223. 10.1080/09658211.2019.1642919. 31307283

[r62] Heerey, E. A., Matveeva, T. M., & Gold, J. M. (2011). Imagining the future: Degraded representations of future rewards and events in schizophrenia. Journal of Abnormal Psychology, 120(2), 483–489. 10.1037/a0021810. 21171727 PMC3091996

[r63] Herold, C. J., Lässer, M. M., Schmid, L. A., Seidl, U., Kong, L., Fellhauer, I., … Schröder, J. (2013). Hippocampal volume reduction and autobiographical memory deficits in chronic schizophrenia. Psychiatry Research: Neuroimaging, 211(3), 189–194. 10.1016/j.pscychresns.2012.04.002. 23158776

[r64] Herold, C. J., Lässer, M. M., & Schröder, J. (2023). Autobiographical memory impairment in chronic schizophrenia: Significance and clinical correlates. Journal of Neuropsychology, 17(1), 89–107. 10.1111/jnp.12288. 36065152

[r65] Hester, R., Nestor, L., & Garavan, H. (2009). Impaired error awareness and anterior cingulate cortex hypoactivity in chronic cannabis users. Neuropsychopharmacology: official publication of the American College of Neuropsychopharmacology, 34(11), 2450–2458. 10.1038/npp.2009.67. 19553917 PMC2743772

[r66] Hilland, E., Johannessen, C., Jonassen, R., Alnæs, D., Jørgensen, K. N., Barth, C., Andreou, D., Nerland, S., Wortinger, L. A., Smelror, R. E., Wedervang-Resell, K., Bohman, H., Lundberg, M., Westlye, L. T., Andreassen, O. A., Jönsson, E. G., & Agartz, I. (2022). Aberrant default mode connectivity in adolescents with early-onset psychosis: A resting state fMRI study. NeuroImage: Clinical, 33, 102881. 10.1016/j.nicl.2021.102881. 34883402 PMC8662331

[r67] Holt, D. J., Cassidy, B. S., Andrews-Hanna, J. R., Lee, S. M., Coombs, G., Goff, D. C., … Moran, J. M. (2011). An anterior-to-posterior shift in midline cortical activity in schizophrenia during self-reflection. Biological Psychiatry, 69(5), 415–423. 10.1016/j.biopsych.2010.10.003. 21144498 PMC3740539

[r68] Irwin, H. J., Green, M. J., & Marsh, P. J. (1999). Dysfunction in smooth pursuit eye movements and history of childhood trauma. Perceptual and Motor Skills, 89(3 Pt 2), 1230–1236. 10.2466/pms.1999.89.3f.1230. 10710773

[r69] Jones, B. A., Landes, R. D., Yi, R., & Bickel, W. K. (2009). Temporal horizon: Modulation by smoking status and gender. Drug and Alcohol Dependence, 104(Suppl 1), S87–S93. 10.1016/j.drugalcdep.2009.04.001 19446407 PMC2732767

[r70] Jun, S., Miao, D., & Ying, J. (2025). A systematic review and meta-analysis on effect of metacognitive training on cognitive biases in patients with schizophrenia: Implications for psychiatric nursing care. Early Intervention in Psychiatry, 19(4), e70026. 10.1111/eip.70026. 40216439

[r71] Kalantar-Hormozi, B., & Mohammadkhani, S. (2024). Reported history of childhood trauma, mentalizing deficits, and hypersomnia in adulthood: A mediational analysis in a nonclinical sample. Brain and Behavior, 14(1), e3363. 10.1002/brb3.3363. 38376014 PMC10761325

[r72] Kansal, V., Patriciu, I., & Kiang, M. (2014). Illness insight and neurophysiological error-processing deficits in schizophrenia. Schizophrenia Research, 156(1), 122–127. 10.1016/j.schres.2014.03.023. 24739490

[r73] Kelly, S., Jahanshad, N., Zalesky, A., Kochunov, P., Agartz, I., Alloza, C., … Donohoe, G. (2018). Widespread white matter microstructural differences in schizophrenia across 4322 individuals: Results from the ENIGMA schizophrenia DTI working group. Molecular Psychiatry, 23(5), 1261–1269. 10.1038/mp.2017.170. 29038599 PMC5984078

[r74] Konsztowicz, S., Schmitz, N., & Lepage, M. (2018). Dimensions of insight in schizophrenia: Exploratory factor analysis of items from multiple self- and interviewer-rated measures of insight. Schizophrenia Research, 199, 319–325. 10.1016/j.schres.2018.02.055. 29530378

[r75] Kühn, S., & Gallinat, J. (2013). Resting-state brain activity in schizophrenia and major depression: A quantitative meta-analysis. Schizophrenia Bulletin, 39(2), 358–365. 10.1093/schbul/sbr151. 22080493 PMC3576173

[r76] Lee, S., Parthasarathi, T., & Kable, J. W. (2021). The ventral and dorsal default mode networks are Dissociably modulated by the vividness and valence of imagined events. Journal of Neuroscience, 41(24), 5243–5250. 10.1523/JNEUROSCI.1273-20.2021. 34001631 PMC8211541

[r77] Leonhardt, B. L., Vohs, J. L., Bartolomeo, L. A., Visco, A., Hetrick, W. P., Bolbecker, A. R., … O’Donnell, B. F. (2020). Relationship of metacognition and insight to neural synchronization and cognitive function in early phase psychosis. Clinical EEG and Neuroscience, 51(4), 259–266. 10.1177/1550059419857971. 31241355

[r78] Lincoln, T. M., Lüllmann, E., & Rief, W. (2007). Correlates and long-term consequences of poor insight in patients with schizophrenia. A systematic review. Schizophrenia Bulletin, 33(6), 1324–1342. 10.1093/schbul/sbm002. 17289653 PMC2779879

[r79] Livingston, N. R., Kiemes, A., O’Daly, O., Knight, S. R., Lukow, P. B., Jelen, L. A., **…** Modinos, G. (2024). Diazepam modulates hippocampal CA1 functional connectivity in people at clinical high-risk for psychosis. medRxiv. 10.1101/2024.12.20.24319330. PMC12360692 40776396

[r80] López-Morínigo, J. D., Martínez, A. S., Barrigón, M. L., Escobedo-Aedo, P. J., Ruiz-Ruano, V. G., Sánchez-Alonso, S., … David, A. S. (2023). A pilot 1-year follow-up randomised controlled trial comparing metacognitive training to psychoeducation in schizophrenia. Effects on insight. Schizophrenia, 9(1), 7. 10.1038/s41537-022-00316-x. 36717598 PMC9886217

[r81] Lungu, P. F., Lungu, C.-M., Ciobîcă, A., Balmus, I. M., Boloș, A., Dobrin, R., & Luca, A. C. (2023). Metacognition in schizophrenia Spectrum disorders-current methods and approaches. Brain Sciences, 13(7), 1004. 10.3390/brainsci13071004. 37508936 PMC10377717

[r82] Luther, L., Bonfils, K. A., Fischer, M. W., Johnson-Kwochka, A. V., & Salyers, M. P. (2020). Metacognition moderates the relationship between self-reported and clinician-rated motivation in schizophrenia. Schizophrenia Research: Cognition, 19, 100140. 10.1016/j.scog.2019.100140. 31828017 PMC6889663

[r83] Lysaker, P. H., Carcione, A., Dimaggio, G., Johannesen, J. K., Nicolò, G., Procacci, M., & Semerari, A. (2005). Metacognition amidst narratives of self and illness in schizophrenia: Associations with neurocognition, symptoms, insight and quality of life. Acta Psychiatrica Scandinavica, 112(1), 64–71. 10.1111/j.1600-0447.2005.00514.x. 15952947

[r84] Lysaker, P. H., Chernov, N., Moiseeva, T., Sozinova, M., Dmitryeva, N., Alyoshin, V., … Kostyuk, G. (2021). Clinical insight, cognitive insight and metacognition in psychosis: Evidence of mediation. Journal of Psychiatric Research, 140, 1–6. 10.1016/j.jpsychires.2021.05.030. 34087750

[r85] Lysaker, P. H., & Dimaggio, G. (2014). Metacognitive capacities for reflection in schizophrenia: Implications for developing treatments. Schizophrenia Bulletin, 40(3), 487–491. 10.1093/schbul/sbu038. 24636965 PMC3984530

[r86] Lysaker, P. H., Gagen, E., Moritz, S., & Schweitzer, R. D. (2018). Metacognitive approaches to the treatment of psychosis: A comparison of four approaches. Psychology Research and Behavior Management, 11, 341–351. 10.2147/PRBM.S146446. 30233262 PMC6130286

[r87] Lysaker, P. H., Gagen, E., Wright, A., Vohs, J. L., Kukla, M., Yanos, P. T., & Hasson-Ohayon, I. (2019). Metacognitive deficits predict impaired insight in schizophrenia across symptom profiles: A latent class analysis. Schizophrenia Bulletin, 45(1), 48–56. 10.1093/schbul/sby142. 30321433 PMC6293218

[r88] Lysaker, P. H., Pattison, M. L., Leonhardt, B. L., Phelps, S., & Vohs, J. L. (2018). Insight in schizophrenia spectrum disorders: Relationship with behavior, mood and perceived quality of life, underlying causes and emerging treatments. World Psychiatry, 17(1), 12–23. 10.1002/wps.20508. 29352540 PMC5775127

[r89] MacDougall, A. G., McKinnon, M. C., Herdman, K. A., King, M. J., & Kiang, M. (2015). The relationship between insight and autobiographical memory for emotional events in schizophrenia. Psychiatry Research, 226(1), 392–395. 10.1016/j.psychres.2014.12.058. 25623015

[r90] Martiadis, V., Pessina, E., Raffone, F., Iniziato, V., Martini, A., & Scognamiglio, P. (2023). Metacognition in schizophrenia: A practical overview of psychometric metacognition assessment tools for researchers and clinicians. Frontiers in Psychiatry, 14. 10.3389/fpsyt.2023.1155321. PMC10133516 37124248

[r91] Martin, A. M. S., Bullock, J., Fiszdon, J., Stacy, M., Martino, S., James, A. V., & Lysaker, P. H. (2023). A guide for the implementation of group-based metacognitive reflection and insight therapy (MERITg). Journal of Contemporary Psychotherapy: On the Cutting Edge of Modern Developments in Psychotherapy, 53(1), 91–98. 10.1007/s10879-022-09560-9.

[r92] Mavrogiorgou, P., Thomaßen, T., Pott, F., Flasbeck, V., Steinfath, H., & Juckel, G. (2022). Time experience in patients with schizophrenia and affective disorders. European Psychiatry, 65(1), e11. 10.1192/j.eurpsy.2022.2. 35094726 PMC8853857

[r93] McCormick, C., Ciaramelli, E., De Luca, F., & Maguire, E. A. (2018). Comparing and contrasting the cognitive effects of hippocampal and ventromedial prefrontal cortex damage: A review of human lesion studies. Neuroscience, 374, 295–318. 10.1016/j.neuroscience.2017.07.066. 28827088 PMC6053620

[r94] Mediavilla, R., López-Arroyo, M., Gómez-Arnau, J., Wiesepape, C., Lysaker, P. H., & Lahera, G. (2021). Autobiographical memory in schizophrenia: The role of metacognition. Comprehensive Psychiatry, 109, 152254. 10.1016/j.comppsych.2021.152254. 34174693

[r95] Micheloyannis, S. (2012). Graph-based network analysis in schizophrenia. World Journal of Psychiatry, 2(1), 1–12. 10.5498/wjp.v2.i1.1. 24175163 PMC3782171

[r96] Miranda-Dominguez, O., Mills, B. D., Carpenter, S. D., Grant, K. A., Kroenke, C. D., Nigg, J. T., & Fair, D. A. (2014). Connectotyping: Model based fingerprinting of the functional Connectome. PLoS One, 9(11), e111048. 10.1371/journal.pone.0111048. 25386919 PMC4227655

[r97] Moeller, J. R., & Habeck, C. G. (2006). Reciprocal benefits of mass-univariate and multivariate modeling in brain mapping: Applications to event-related functional MRI, H215O-, and FDG-PET. International Journal of Biomedical Imaging, 2006(1), 079862. 10.1155/IJBI/2006/79862. PMC2324050 23165047

[r98] Molnar-Szakacs, I., & Uddin, L. Q. (2013). Self-processing and the default mode network: Interactions with the mirror neuron system. Frontiers in Human Neuroscience, 7, 571. 10.3389/fnhum.2013.00571. 24062671 PMC3769892

[r99] Monk, A. M., Barnes, G. R., & Maguire, E. A. (2020). The effect of object type on building scene imagery—An MEG study. Frontiers in Human Neuroscience, 14, 592175. 10.3389/fnhum.2020.592175. 33240069 PMC7683518

[r100] Moore, M. T., & Fresco, D. M. (2012). Depressive realism: A meta-analytic review. Clinical Psychology Review, 32(6), 496–509. 10.1016/j.cpr.2012.05.004. 22717337

[r101] O’Neill, A., Mechelli, A., & Bhattacharyya, S. (2019). Dysconnectivity of large-scale functional networks in early psychosis: A meta-analysis. Schizophrenia Bulletin, 45(3), 579–590. 10.1093/schbul/sby094. 29982729 PMC6483589

[r102] Østby, Y., Walhovd, K. B., Tamnes, C. K., Grydeland, H., Westlye, L. T., & Fjell, A. M. (2012). Mental time travel and default-mode network functional connectivity in the developing brain. Proceedings of the National Academy of Sciences of the United States of America, 109(42), 16800–16804. 10.1073/pnas.1210627109. 23027942 PMC3479452

[r103] Pagnotta, M. F., Riddle, J., & D’Esposito, M. (2024). Multimodal neuroimaging of hierarchical cognitive control. Biological Psychology, 193, 108896. 10.1016/j.biopsycho.2024.108896. 39488242

[r104] Pan, Y., Liu, Z., Xue, Z., Sheng, Y., Cai, Y., Cheng, Y., & Chen, X. (2022). Abnormal network properties and fiber connections of DMN across major mental disorders: A probability tracing and graph theory study. Cerebral Cortex (New York, N.Y.: 1991), 32(15), 3127–3136. 10.1093/cercor/bhab405 34849632

[r105] Pedrelli, P., McQuaid, J. R., Granholm, E., Patterson, T. L., McClure, F., Beck, A. T., & Jeste, D. V. (2004). Measuring cognitive insight in middle-aged and older patients with psychotic disorders. Schizophrenia Research, 71(2–3), 297–305. 10.1016/j.schres.2004.02.019. 15474900

[r106] Peng, Y., Zhang, S., Zhou, Y., Song, Y., Yang, G., Hao, K., … Zhang, Y. (2021). Abnormal functional connectivity based on nodes of the default mode network in first-episode drug-naive early-onset schizophrenia. Psychiatry Research, 295, 113578. 10.1016/j.psychres.2020.113578. 33243520

[r107] Perrottelli, A., Giordano, G. M., Brando, F., Giuliani, L., Pezzella, P., Mucci, A., & Galderisi, S. (2022). Unveiling the associations between EEG indices and cognitive deficits in schizophrenia-Spectrum disorders: A systematic review. Diagnostics, 12(9), 9. 10.3390/diagnostics12092193. PMC9498272 36140594

[r108] Potheegadoo, J., Cuervo-Lombard, C., Berna, F., & Danion, J.-M. (2012). Distorted perception of the subjective temporal distance of autobiographical events in patients with schizophrenia. Consciousness and Cognition, 21(1), 90–99. 10.1016/j.concog.2011.09.012. 21993451

[r109] Raffard, S., D’Argembeau, A., Bayard, S., Boulenger, J.-P., & Van der Linden, M. (2010). Scene construction in schizophrenia. Neuropsychology, 24(5), 608–615. 10.1037/a0019113. 20804249

[r110] Raffard, S., D’Argembeau, A., Lardi, C., Bayard, S., Boulenger, J.-P., & Van Der Linden, M. (2009). Exploring self-defining memories in schizophrenia. Memory (Hove, England), 17(1), 26–38. 10.1080/09658210802524232. 19105085

[r111] Raffard, S., D’Argembeau, A., Lardi, C., Bayard, S., Boulenger, J.-P., & Van der Linden, M. (2010). Narrative identity in schizophrenia. Consciousness and Cognition, 19(1), 328–340. 10.1016/j.concog.2009.10.005. 19955004

[r112] Redaelli, S., Porffy, L., Oloyede, E., Dzahini, O., Lewis, G., Lobo, M., … Shergill, S. S. (2022). Vortioxetine as adjunctive therapy in the treatment of schizophrenia. Therapeutic Advances in Psychopharmacology, 12, 20451253221110014. 10.1177/20451253221110014. 35833056 PMC9272178

[r113] Riggs, S. E., Grant, P. M., Perivoliotis, D., & Beck, A. T. (2012). Assessment of cognitive insight: A qualitative review. Schizophrenia Bulletin, 38(2), 338–350. 10.1093/schbul/sbq085. 20693342 PMC3283158

[r114] Rung, J. M., & Madden, G. J. (2018). Experimental reductions of delay discounting and impulsive choice: A systematic review and meta-analysis. Journal of Experimental Psychology. General, 147(9), 1349–1381. 10.1037/xge0000462. 30148386 PMC6112163

[r115] Salvador, R., Verdolini, N., Garcia-Ruiz, B., Jiménez, E., Sarró, S., Vilella, E., … Voineskos, A. N. (2020). Multivariate brain functional connectivity through regularized estimators. Frontiers in Neuroscience, 14, 569540. 10.3389/fnins.2020.569540. 33363451 PMC7753183

[r116] Samartzis, L., Dima, D., Fusar-Poli, P., & Kyriakopoulos, M. (2014). White matter alterations in early stages of schizophrenia: A systematic review of diffusion tensor imaging studies. Journal of Neuroimaging: Official Journal of the American Society of Neuroimaging, 24(2), 101–110. 10.1111/j.1552-6569.2012.00779.x. 23317110

[r117] Semerari, A., Carcione, A., Dimaggio, G., Falcone, M., Nicolò, G., Procacci, M., & Alleva, G. (2003). How to evaluate metacognitive functioning in psychotherapy? The metacognition assessment scale and its applications. Clinical Psychology & Psychotherapy, 10(4), 238–261. 10.1002/cpp.362.

[r118] Sendi, M. S. E., Zendehrouh, E., Ellis, C. A., Liang, Z., Fu, Z., Mathalon, D. H., … Calhoun, V. D. (2021). Aberrant dynamic functional connectivity of default mode network in schizophrenia and links to symptom severity. Frontiers in Neural Circuits, 15, 649417. 10.3389/fncir.2021.649417. 33815070 PMC8013735

[r119] Shan, X., Liao, R., Ou, Y., Ding, Y., Liu, F., Chen, J., … He, Y. (2020). Metacognitive training modulates default-mode network homogeneity during 8-week olanzapine treatment in patients with schizophrenia. Frontiers in Psychiatry, 11, 234. 10.3389/fpsyt.2020.00234. 32292360 PMC7118222

[r120] Shirer, W. R., Ryali, S., Rykhlevskaia, E., Menon, V., & Greicius, M. D. (2012). Decoding subject-driven cognitive states with whole-brain connectivity patterns. Cerebral Cortex (New York, N.Y.: 1991), 22(1), 158–165. 10.1093/cercor/bhr099 21616982 PMC3236795

[r121] Simons, J. S., Garrison, J. R., & Johnson, M. K. (2017). Brain mechanisms of reality monitoring. Trends in Cognitive Sciences, 21(6), 462–473. 10.1016/j.tics.2017.03.012. 28462815

[r122] Slifstein, M., van de Giessen, E., Van Snellenberg, J., Thompson, J. L., Narendran, R., Gil, R., … Abi-Dargham, A. (2015). Deficits in prefrontal cortical and extrastriatal dopamine release in schizophrenia: A positron emission tomographic functional magnetic resonance imaging study. JAMA Psychiatry, 72(4), 316–324. 10.1001/jamapsychiatry.2014.2414. 25651194 PMC4768742

[r123] Stanghellini, G., Ballerini, M., Presenza, S., Mancini, M., Raballo, A., Blasi, S., & Cutting, J. (2016). Psychopathology of lived time: Abnormal time experience in persons with schizophrenia. Schizophrenia Bulletin, 42(1), 45–55. 10.1093/schbul/sbv052. 25943123 PMC4681541

[r124] Stevenson, R. A., Park, S., Cochran, C., McIntosh, L. G., Noel, J. P., Barense, M. D., Ferber, S. & Wallace, M. T. (2017). The associations between multisensory temporal processing and symptoms of schizophrenia. Schizophrenia research, 179, 97–103. 10.1016/j.schres.2016.09.035. 27746052 PMC5463449

[r125] Suddendorf, T., & Corballis, M. C. (2007). The evolution of foresight: What is mental time travel, and is it unique to humans? The Behavioral and Brain Sciences, 30(3), 299–313; discussion 313–351. 10.1017/S0140525X07001975 17963565

[r126] Takarangi, M. K. T., Smith, R. A., Strange, D., & Flowe, H. D. (2017). Metacognitive and Metamemory beliefs in the development and maintenance of posttraumatic stress disorder. Clinical Psychological Science, 5(1), 131–140. 10.1177/2167702616649348.

[r127] Tulving, E. (1985). Memory and consciousness. Canadian Psychology/Psychologie Canadienne, 26(1), 1–12. 10.1037/h0080017.

[r128] van der Meer, L., de Vos, A. E., Stiekema, A. P., Pijnenborg, G. H., van Tol, M. J., Nolen, W. A., … Aleman, A. (2013). Insight in schizophrenia: Involvement of self-reflection networks? Schizophrenia Bulletin, 39(6), 1288–1295. 10.1093/schbul/sbs122. PMC3796073 23104865

[r129] Vanasse, T. J., Fox, P. T., Fox, P. M., Cauda, F., Costa, T., Smith, S. M., … Lancaster, J. L. (2021). Brain pathology recapitulates physiology: A network meta-analysis. Communications Biology, 4(1), 301. 10.1038/s42003-021-01832-9. 33686216 PMC7940476

[r130] Vazquez-Trejo, V., Nardos, B., Schlaggar, B. L., Fair, D. A., & Miranda-Dominguez, O. (2022). Use of connectotyping on task functional MRI data reveals dynamic network level cross talking during task performance. Frontiers in Neuroscience, 16, 951907. 10.3389/fnins.2022.951907. 36300171 PMC9589037

[r131] Viard, A., Chételat, G., Lebreton, K., Desgranges, B., Landeau, B., de La Sayette, V., … Piolino, P. (2011). Mental time travel into the past and the future in healthy aged adults: An fMRI study. Brain and Cognition, 75(1), 1–9. 10.1016/j.bandc.2010.10.009. 21093970

[r132] Vincent, J. L., Kahn, I., Snyder, A. Z., Raichle, M. E., & Buckner, R. L. (2008). Evidence for a frontoparietal control system revealed by intrinsic functional connectivity. Journal of Neurophysiology, 100(6), 3328–3342. 10.1152/jn.90355.2008. 18799601 PMC2604839

[r133] Vohs, J. L., Lysaker, P. H., Francis, M. M., Hamm, J., Buck, K. D., Olesek, K., … Breier, A. (2014). Metacognition, social cognition, and symptoms in patients with first episode and prolonged psychoses. Schizophrenia Research, 153(1–3), 54–59. 10.1016/j.schres.2014.01.012. 24503175

[r134] Vohs, J. L., Lysaker, P. H., Liffick, E., Francis, M. M., Leonhardt, B. L., James, A., … Breier, A. (2015). Metacognitive capacity as a predictor of insight in first-episode psychosis. The Journal of Nervous and Mental Disease, 203(5), 372–378. 10.1097/NMD.0000000000000291. 25900550

[r135] Von Der Heide, R. J., Skipper, L. M., Klobusicky, E., & Olson, I. R. (2013). Dissecting the uncinate fasciculus: Disorders, controversies and a hypothesis. Brain, 136(6), 1692–1707. 10.1093/brain/awt094. 23649697 PMC3673595

[r136] Weber, S., Johnsen, E., Kroken, R. A., Løberg, E. M., Kandilarova, S., Stoyanov, D., … Hugdahl, K. (2020). Dynamic functional connectivity patterns in schizophrenia and the relationship with hallucinations. Frontiers in Psychiatry, 11, 227. 10.3389/fpsyt.2020.00227. 32300313 PMC7145395

[r137] Weiss-Cowie, S., Verhaeghen, P., & Duarte, A. (2023). An updated account of overgeneral autobiographical memory in depression. Neuroscience and Biobehavioral Reviews, 149, 105157. 10.1016/j.neubiorev.2023.105157. 37030646

[r138] Xu, J., Calhoun, V. D., Worhunsky, P. D., Xiang, H., Li, J., Wall, J. T., … Potenza, M. N. (2015). Functional network overlap as revealed by fMRI using sICA and its potential relationships with functional heterogeneity, balanced excitation and inhibition, and sparseness of neuron activity. PLoS One, 10(2), e0117029. 10.1371/journal.pone.0117029. 25714362 PMC4340936

[r139] Xu, L., Zhang, M., Wang, S., Wei, Y., Cui, H., Qian, Z., … Wang, J. (2021). Relationship between cognitive and clinical insight at different durations of untreated attenuated psychotic symptoms in high-risk individuals. Frontiers in Psychiatry, 12, 753130. 10.3389/fpsyt.2021.753130. 34867540 PMC8637962

[r140] Ye, J. Y., Ding, Q. Y., Cui, J. F., Liu, Z., Jia, L. X., Qin, X. J., … Wang, Y. (2022). A meta-analysis of the effects of episodic future thinking on delay discounting. Quarterly Journal of Experimental Psychology, 75(10), 1876–1891. 10.1177/17470218211066282. 34841982

[r141] You, W., Luo, L., Yao, L., Zhao, Y., Li, Q., Wang, Y., … Li, F. (2022). Impaired dynamic functional brain properties and their relationship to symptoms in never treated first-episode patients with schizophrenia. Schizophrenia, 8(1), 90. 10.1038/s41537-022-00299-9. 36309537 PMC9617869

[r142] Zhang, Y., Kuhn, S. K., Jobson, L., & Haque, S. (2019). A review of autobiographical memory studies on patients with schizophrenia spectrum disorders. BMC Psychiatry, 19(1), 361. 10.1186/s12888-019-2346-6. 31727046 PMC6857214

[r501] Zhang, H., Wang, Y., Hu, Y., Zhu, Y., Zhang, T., Wang, J., & Li, C. (2019). Metaanalysis of cognitive function in Chinese first-episode schizophrenia: MATRICS consensus cognitive battery (MCCB) profile of impairment. General Psychiatry, 32(3), e100043. 10.1136/gpsych-2018-100043. 31423473 PMC6677937

